# Synthesis and Characterization of Natural Polymeric Membranes Composed of Chitosan, Green Banana Peel Extract and Andiroba Oil

**DOI:** 10.3390/polym14061105

**Published:** 2022-03-10

**Authors:** Elisângela da Silva Ferreira, Sheila Barbosa Paranhos, Simone Patrícia Aranha da Paz, Caio Augusto de Almeida Canelas, Luís Adriano Santos do Nascimento, Marcele Fonseca Passos, Alisson Clay Rios da Silva, Sergio Neves Monteiro, Marcos Vinícius da Silva Paula, Verônica Scarpini Candido

**Affiliations:** 1 Engineering of Natural Resources of the Amazon Program, Federal University of Pará—UFPA, Rua Augusto Corrêa 01, Belém, Pará 66075-110, Brazil; licalipe8@yahoo.com.br (E.d.S.F.); paranhos@ufpa.br (S.B.P.); paz@ufpa.br (S.P.A.d.P.); 2 Laboratory of Amazon Oils, Federal University of Pará—UFPA, Augusto Corrêa Street, Belém, Pará 66075-110, Brazil; caio.a.canelas@gmail.com (C.A.d.A.C.); adrlui1@yahoo.com.br (L.A.S.d.N.); 3 Materials Science and Engineering Program, Federal University of Pará, Belém-Pa. Tv We 26, Ananindeua, Pará 67130-660, Brazil; cellepassos@ufpa.br (M.F.P.); alissonrios@ufpa.br (A.C.R.d.S.); 4 Department of Materials Science, Military Institute of Engineering—IME, Praça General Tibúrcio 80, Urca, Rio de Janeiro 22290-270, Brazil; snevesmonteiro@gmail.com; 5 Materials Engineering Faculty, Federal University of Pará, Belém-Pa. Tv We 26, Ananindeua, Pará 67130-660, Brazil; mpaula@ufpa.br

**Keywords:** chitosan, green banana peel extract, andiroba oil, synthesis, technical characterization, biomedical potential

## Abstract

Chitosan comprises polymeric macromolecules with technical and biological properties that have been used in biomedical healing applications requiring anti-microbial and anti-inflammatory capacities worldwide. In the tropical regions, green banana peel extract and andiroba oil are considered natural products with wound healing properties. The present study, for the first time, synthesized chitosan/green banana peel extract/andiroba oil (CGA) membranes and analyzed them using scanning electron microscopy (SEM) and the swelling and moisture tests. The CGA membranes together with control membranes of plain chitosan and chitosan plus green banana peel extract, were characterized by contact angle measurement, X-ray diffraction (XRD) and differential scanning calorimetry (DSC). Macroscopic analysis showed significant differences in color and transparency caused by the number of decoction days used for extract preparation and the oil content. SEM observations disclosed the formation of two phases, lipid and polymer, in the CGA. The number of decoction days and the andiroba oil content were inversely related to the swelling moisture uptake. All membranes were found to be hydrophilic with contact angles less than 90°. The incorporation of plant extract and oil promoted the appearance of related XRD peaks. DSC curves revealed a reduction in the enthalpy of the CGA membranes compared with plain chitosan, which might be attributed to the evaporation of the natural extract and oil. Based on these findings, the studied newly synthesized membranes demonstrated a potential for healing epithelial lesions.

## 1. Introduction

Biomaterials intended for biomedical applications must have properties that make them capable of performing a specific function to compensate for the loss of corresponding applications ones in diseased or damaged tissues. Their study in tissue regeneration has shown satisfactory and unexpected results [1], among them stand those of membranes composed of chitosan.

Chitosan performs many biological activities, such as antimicrobial and anti-inflammatory activities, as well as acting as a therapeutic polymer [2]. In addition to its biocompatibility, biodegradability, non-toxicity, and antimicrobial activity, chitosan provides accelerated wound healing [3].

The properties of chitosan are related to its physical-chemical characteristics. Indeed, when soluble in an acid medium, it becomes positively charged and has high bioactivity. The gains with the functionality of the chitosan macromolecules are the potential of mucus adhesiveness and its interaction with several negatively-charged molecules, wall or cell membrane of microorganisms, proteins, and other epithelial constituents [4].

The association of natural polymers with banana peel has been the object of several studies [4,5] and the related biomaterials have been used to treat skin lesions. In fact, the peel of the green fruit has antimicrobial activity and antioxidant power [4,5]. These healing properties are associated with the presence of minerals like calcium, potassium, magnesium and sodium, as well as phenolic compounds, especially flavonoids and tannins, being present in greater quantity in the green fruit [6,7,8]. Flavonoids are one of the most important phenolic groups and are formed by benzene and pyran rings. Gallocatechin, a type of flavonoid, shows strong antioxidant activity [9]. Tannins are high molecular weight (500–3000 Da) phenolic compounds derived from the plants’ secondary metabolism [9]. These compounds are highly reactive, which leads to protein precipitation, forming a complex that protects the underlying skin and mucus. In addition, these bind to the cell wall of bacteria and fungi, precipitating them and ensuring a more septic environment, allowing healing [8].

Franco et al. [8] pointed out and characterized chitosan membranes associated with banana peel extract. In their results, the non-toxicity to dermal fibroblasts of the material suggested it to have biomedical potential in the wound area.

However, a biomaterial requires particular properties and a single material may not provide desirable in vivo characteristics [10]. This work shows the importance of improving these properties by the addition of andiroba oil in membrane synthesis.

Souza et al. [11] have shown that andiroba oil effectively participates in wound healing, promoting the formation of granulation tissue, tissue contraction, and epithelialization. Several other studies have identified that andiroba seeds are rich in limonoids, which confer a variety of biological activities, such as antimalarial, antiallergic, anti-inflammatory and antioxidant effects, conferring therapeutic and medicinal properties [12,13,14].

Consider the limitations of current therapies. It is necessary to develop and evaluate new biomaterials that contribute to the healing and regeneration of functional tissues [10]. In this context, the aim of this study is to analyze the influence of the addition of green banana peel extract and different concentrations of andiroba oil on the properties of chitosan membranes. It is worth mentioning that this is the first time that these three components have been associated for the production of a biomaterial. Consequently, it is the first time that the physical, chemical and morphological characterization of these membranes was carried out. Moreover, the influence of the addition of two different concentrations of andiroba oil on the physical and chemical properties of the membranes has not been studied before. In addition, it is believed that the concentrations studied might favor the production of more flexible membranes with absorption and swelling capacity, which are desirable characteristics in materials used as dressings for medical applications.

## 2. Materials and Methods

### 2.1. Materials

For the manufacture of membranes, the materials described in Table 1 were used.

### 2.2. Preparation of Green Banana Peel Extracts by Decoction

The green banana peels were removed from the fruits (250 g) and were disinfected by immersion in sodium hypochlorite solution 1% *v*/*v* for 15 min. They were then washed in distilled water, dried on paper towels, cut into cubes (1 cm^3^), boiled in 1 L of distilled water for 2 h on a heating plate, and then packed in an amber glass jar and stored under refrigeration (approximately 4 °C) for 24 h. The next day, the product was filtered in a nylon filter to obtain the extract of the first day. The peels were reused, following the process described in the preparation of the extracts, for the second and third days.

### 2.3. Synthesis of Membranes

**Figure 1:**
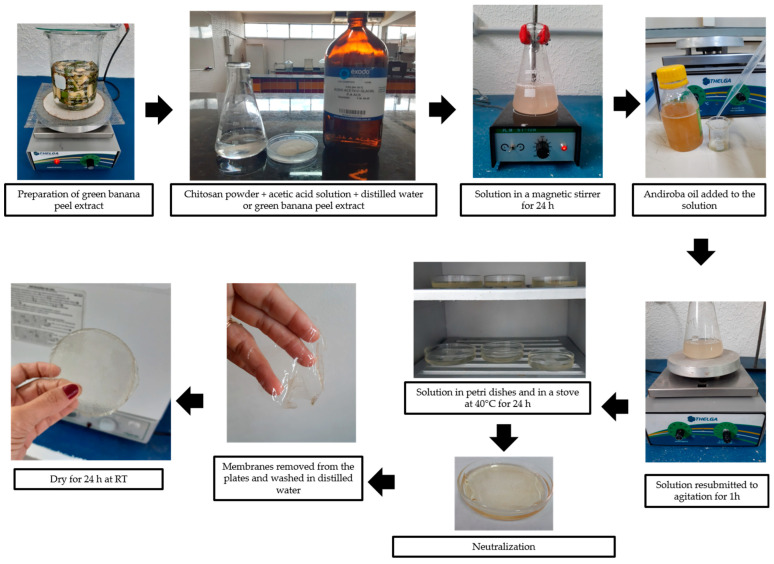
Schematic diagram of the synthesis of membranes by the casting method.

The preparation of the emulsions used in the synthesis of membranes followed the method described by Franco et al. [8], with modifications. The membranes were obtained by the casting method illustrated in Figure 1. The chitosan powder (1% *w*/*v*) was dissolved in an aqueous solution of acetic acid (1% *v*/*v*), distilled water or green banana peel extract (100 mL), according to membrane composition. The solution was maintained in a magnetic stirrer for 24 h at room temperature (RT). Andiroba oil was added to the solution at two different concentrations (0.1 mL and 0.5 mL) and it was resubjected to agitation for 1 h.

Each solution was melted in polystyrene petri dishes of 8 cm in diameter (50 mL in each) and placed in a stove at 40° C for 24 h. Dry membranes were neutralized with 5% *v*/*v* sodium hydroxide solution for 2 h. After that, the membranes were washed with distilled water to remove the excess from the reagent and dried for 24 h at RT.

The novel membranes, composed of chitosan, green banana peel extract and andiroba oil were named CGA, for short. The other types of synthesized membranes were:

(i) Plain chitosan;
(ii) Chitosan plus green banana peel extract; and
(iii) Chitosan plus andiroba oil (studied as control samples).

Table 2 summarizes the composition of the different samples of synthesized membranes.

### 2.4. Macroscopic Analysis

The macroscopic analysis was performed by visual observation of the membranes regarding the aspects of color, uniformity, transparency, the presence or absence of bubbles and cracks, brittle appearance, flexibility and detachment of the support by means of scale parameters, where zero (0) means absence and three (3) is the maximum score.

### 2.5. Swelling Test

Samples of dry membranes (Wd) were previously weighed and immersed in water and PBS at RT for different time intervals (1, 2, 3, 4, 5, 24, 168, and 336 h). After each period, the samples were removed and cleaned with absorbent paper to remove the excess liquid adhered to the surface. The wet weight (Ws) was determined using a Chyo digital analytical balance, model JK 200. The percentage of swelling (S) was calculated as:

(1) $$S\left(\%\right)=\frac{\left(\mathrm{Ws}-\mathrm{Wd}\right)}{\left(\mathrm{Wd}\right)}\times 100$$

### 2.6. Moisture Test

The percentage of moisture (M) of the membranes was determined using the gravimetric method by weighing the initial mass (Wi) and the final mass (Wf) after 24 h in a stove at 105 °C, according to:

(2) $$M\left(\%\right)=\frac{\left(\mathrm{Wi}-\mathrm{Wf}\right)}{\left(\mathrm{Wi}\right)}\times 100$$

### 2.7. Contact Angle

To evaluate the hydrophilicity of the membranes, the sessile drop method was used by applying a drop of distilled water on the samples of the synthesized membranes. The images were captured by a camera and transferred to Image J software, version 1.51n (National Institute Health, New York, NY, USA) to measure the contact angle.

### 2.8. Scanning Electron Microscopy (SEM)

Morphological analysis was performed through scanning electron microscopy (SEM) using a Tescan-Vega 3 microscope (Kohoutovice, Czech Republic)**,** equipped with a 5 kV field emission electron source.

Surface images of the membranes were obtained with different magnifications. The samples were fractured in liquid nitrogen, mounted on a metal smear and coated with a thin layer of gold–palladium.

### 2.9. X-ray Diffraction (XRD)

PANalytical XRD analyses were performed using an Empyrean Model X-ray diffractometer (Malvern, United Kingdom) using Co-anode ceramic X-ray tubes (Kα1 = 1.789010 Å). Phase identification was performed using X-Pert HighScore Plus (Panalytical) software (Malvern Panalytical, version 5.1).

### 2.10. Differential Scanning Calorimetry (DSC)

The stability of membranes against temperature variations was evaluated using the differential scanning calorimetry (DSC) technique employing a Shimadzu DSC-60 thermogravimetric analyzer (Kyoto, Japan). About 5 mg of each sample was placed in a closed aluminum container. Samples were heated from 25 °C to 250 °C, then they underwent an isotherm phase of 250 °C for 5 min, cooling from 250 °C to 25 °C, an isotherm phase of 25 °C for 5 min and heating from 25 °C to 550 °C, with all temperature variations occurring at a rate of 10 °C/min, under a nitrogen flow of 50 mL/min.

### 2.11. Statistical Analysis

The results of the swelling, moisture and angle of membrane contact tests were expressed as means and standard deviation, and evaluated using variance analysis (ANOVA), with significance verified with the Tukey test at 95% (*p* < 0.05).

## 3. Results and Discussion

### 3.1. Macroscopic Analysis

**Figure 2:**
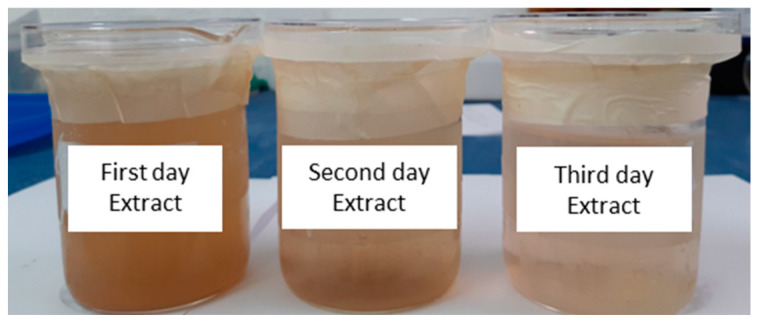
Extracts from green banana peel prepared by the decoction method.

The visual aspect of green banana peel extract is presented in Figure 2. The extracts of the green banana peel showed a brownish color, and the first day decoction was cloudiest and darker than the others. The extract from the third day of decoction was more transparent.

The darkening of the solution can be attributed to the enzymatic oxidation of the tannins present in the banana peel [8]. Therefore, it is suggested that there is a large amount of tannic acids in the more brownish solution (extract from the first decoction).

The synthesized membranes presented different aspects regarding color, uniformity, transparency, presence of bubbles/cracks, brittle appearance, flexibility, and detachment of the support. The characteristics of the membranes are indicated in Table 3.

**Figure 3:**
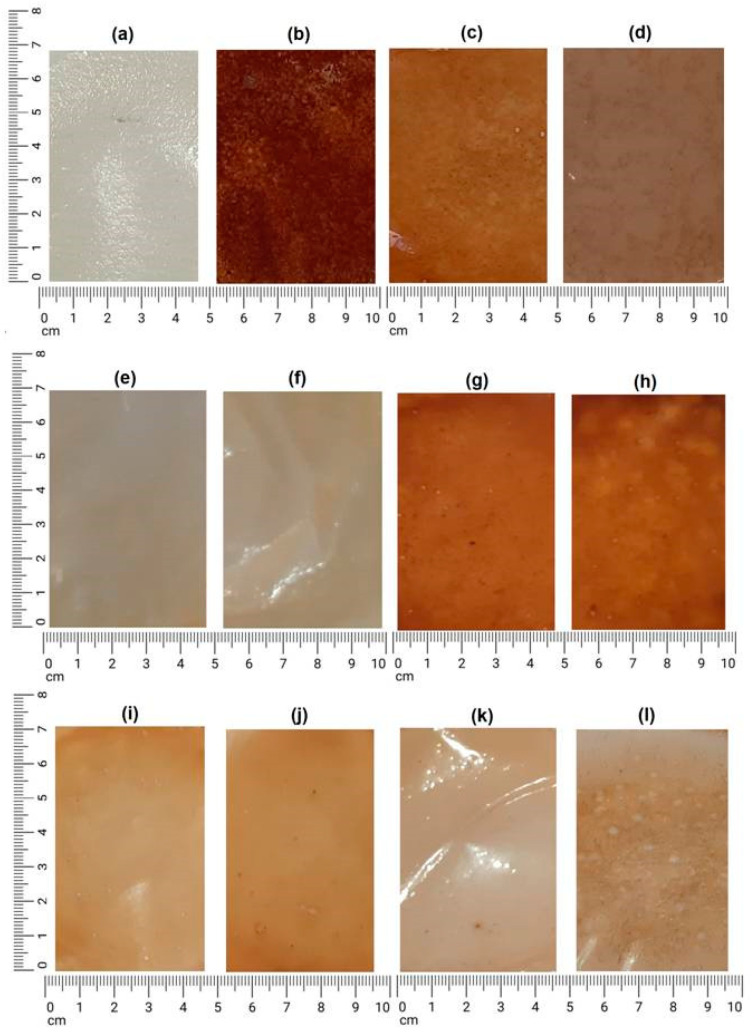
Membranes of synthesized chitosan. (**a**) M0. (**b**) M1. (**c**) M2. (**d**) M3. (**e**) M4. (**f**) M5. (**g**) M6. (**h**) M7. (**i**) M8. (**j**) M9. (**k**) M10. (**l**) M11.

It was observed that the visual aspect of each membrane was related to the components of its synthesis. Greater amounts of darkening and opacity were associated with the products composed of green banana peel extract from the first day of decoction and greater translucency of the membranes occurred in the products with no added green banana peel extract (M0, M4, and M5). The macroscopic visual staining did not depend on the concentration of added andiroba oil (0.1 mL or 0.5 mL). It was noticed that the green banana peel extract influences the brownish staining of the membranes (see Figure 3).

The higher the concentration of andiroba oil, the more difficult it was to incorporate into the membrane, owing to drying and evaporation. In addition, the oily aspect to the touch changed in association with a relationship directly proportional to the amount of oil added to the solution.

There is a color difference between the pure chitosan membranes (M0) and those containing andiroba oil, even without adding green banana peel extract. Therefore, it is perceived that the brownish color action of andiroba oil also influenced the color of the M4 and M5 membranes.

### 3.2. Swelling and Moisture Test

Table 4 shows that the percentage of moisture of membranes composed of green banana peel extract was higher, which indicates the higher hydrophilic character of phenolic compounds when compared to chitosan and andiroba oil.

Retention and absorption capacity are considered essential parameters for evaluating the quality of absorbent dressings [16]. Swelling is directly related to absorbance capacity and is considered a decisive factor for the adequate diffusion of cells and nutrients. Indeed, a high degree of swelling might favor the healing process [17].

There was a statistically significant difference in the percentage of swelling between the membranes and the used solutions, water and PBS (ANOVA, *p* > 0.005) with *p* = 0.00603 (F 5.08 > F critical 2.81) and *p* = 0.032621 (F 5.97 > F critical 4.84) respectively.

The membranes with the highest percentage of swelling were prepared with chitosan and andiroba oil (M4 and M5), without green banana peel extract in their composition.

The results of this study point to an inversely proportional relationship between swelling and the moisture of the synthesized membranes. This behavior might be explained by the presence of hydroxyl and amine groups in chitosan that may interact with water, increasing the swelling capacity of the membranes.

Similar results to this study were found by Franco et al. [8], who did not observe a significant difference in the moisture content of chitosan membranes and green banana peel extracts, suggesting that the presence of extracted compounds does not influence the water content in the biomaterial.

However, Zhang et al. [18] pointed out that the lower moisture content of chitosan membranes with green banana peel extract was associated with the presence of phenolic substances in the extract. These authors suggest that the hydroxyl and carboxyl groups of the phenolic group increase their binding capacity to the chitosan molecule, reducing their binding to water molecules.

The concentration of andiroba oil in chitosan membranes decreases the moisture loss capacity of the material due to the hydrophobicity of the oil [19]. It is noteworthy that a high percentage of moisture can provide propitious means for microorganism proliferation and membrane degradation, culminating in a less viable and effective biomedical material.

In this study, all membranes presented significant swelling capacity, which may be related to the presence of chitosan in their composition. The water uptake power of chitosan is related to the hydrophilic groups (hydroxyl and amino group) of the polysaccharide, characterized by covalent bonds (N-H). In fact, the electronegativity of the bonds generates sites of high polarity, thus producing a favorable rearrangement of water molecules around such sites, characterizing the material as one with a high degree of affinity and water retention [4,20].

**Figure 4:**
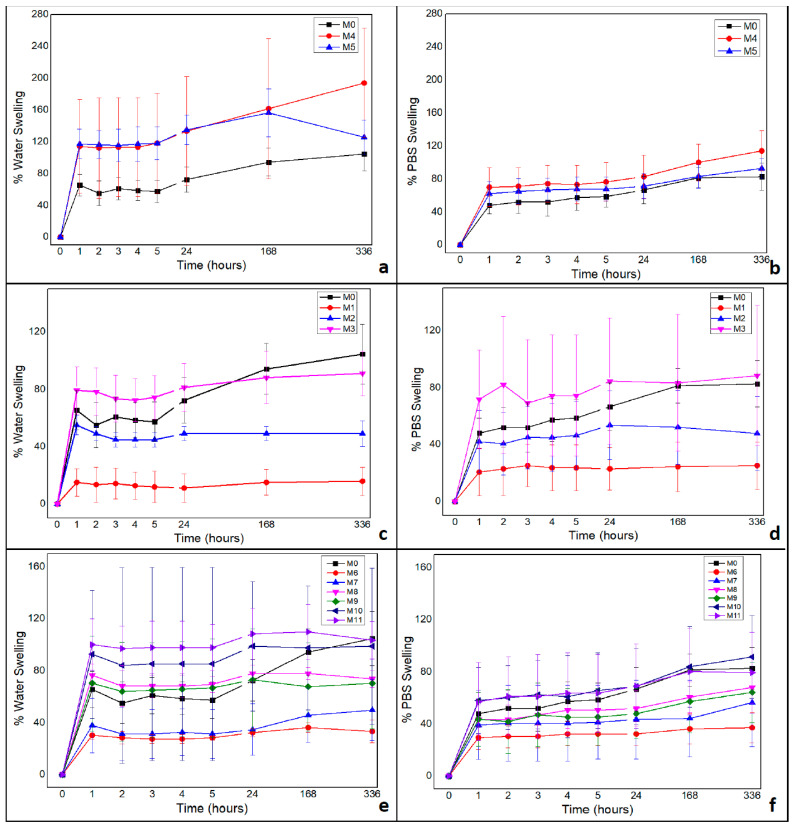
The membranes composed of chitosan and andiroba oil swelling in water (**a**) in PBS (**b**); the percentage of swelling of the membranes containing green banana peel extract from the first and second decoction days (M1 and M2) respectively in water (**c**) and in PBS (**d**); the percentage of swelling of the membranes containing green banana peel extract from the third day of decoction (M10 and M11) in water (**e**) and in PBS (**f**).

It was observed that there was a continuous increase in the percentage of swelling over the time evaluated for all membranes, regardless of composition, as demonstrated in Figure 4.

By and large, the membranes presented a higher percentage of swelling in water and a lower percentage in PBS. This happened in all periods of analysis, except for membranes prepared with the green banana peel extract decoction of the first day (M1, M6, and M7), which had higher swelling in PBS in most of the analyzed hours.

Figure 4 demonstrates that these membranes presented a swelling greater than that of the pure chitosan membrane, with no statistically significant difference between M4 and M5, both in water (ANOVA, *p* > 0.005 = 0.27; F 1.26 < F critical 4.28) and in PBS (ANOVA, *p* > 0.005 = 0.11; F 2.76 < F critical 4.28).

As for membranes composed of chitosan and andiroba oil, it was observed that the swelling in water (a) was more pronounced than in PBS (b), was continuous in both conditions throughout the evaluated time, with stability after 7 days (168 h), except for the M5 membrane in water, which after this period showed a decrease in the percentage of swelling.

In Figure 4, it can also be noted that the membranes containing green banana peel extract from the first and second decoction days (M1 and M2, respectively) presented a percentage of swelling lower than M0 both in water (c) and in PBS (d), which might be indicative of strong interactions between the chitosan matrix and the compounds extracted from the green banana peel, decreasing the water absorption capacity.

There was a relationship between the percentage of swelling and the concentration of green banana peel extract, even with the addition of andiroba oil. Actually, there was a smaller swelling of membranes prepared with the decoction extract of the first day (M6 and M7), followed by membranes prepared with the extract of the second day (M8 and M9). Moreover, a higher percentage of materials synthesized with green banana peel extract from the third day of decoction (M10 and M11), occurred in water (e) and PBS (f).

The incorporation of andiroba oil in the chitosan membrane promoted a change in fluid absorption in the swelling test. On the other hand, the membranes prepared with green banana peel extract of the decoction of the first day presented the lowest percentages of swelling, followed by the extracts from the second day and the third day, with continuous growth over the evaluated time and up to 24 h, with stability after this period.

A similar result was found in the study by Franco et al. [8], reporting that water uptake was inversely proportional to the concentration of banana peel extract, in which the membrane prepared with the decoction extract of the first day exhibited lower swelling due to the high concentration of phenolic compounds. The authors suggested that the active compounds of banana peel interacted with the polymer matrix, reducing water absorption due to chitosan interactions and hydroxyl grouping (—OH).

In the study by Kamel et al. [5] nanocomposites were prepared and characterized from chitosan and banana peel powder at different concentrations. Their results of the swelling test indicated that the addition of banana peel powder in the material resulted in a decrease in the percentage of swelling. This behavior might have been attributed to some interaction between negatively charged compounds in the banana peel, such as carboxylic acids, and positively charged groups in the structure of chitosan, restricting the mobility of chitosan chains and decreasing water absorption.

The addition of andiroba oil provides greater absorption power to membranes which influence the lipid compounds characteristic of oily components. Owing to this, there was a balance in the swelling of membranes composed of vegetal oil.

This fact might be ideal if considering the importance of a biomaterial with excessive absorbent capacity, since the ideal in the healing process is that the epithelial lesions remain moist. While an absorbing material becomes a healing facilitator, excess moisture might favor the proliferation of unwanted microorganisms that can lead to localized infections. As such, it is necessary to synthesize a biomaterial with balance in the absorption process.

### 3.3. Contact Angle

**Figure 5:**
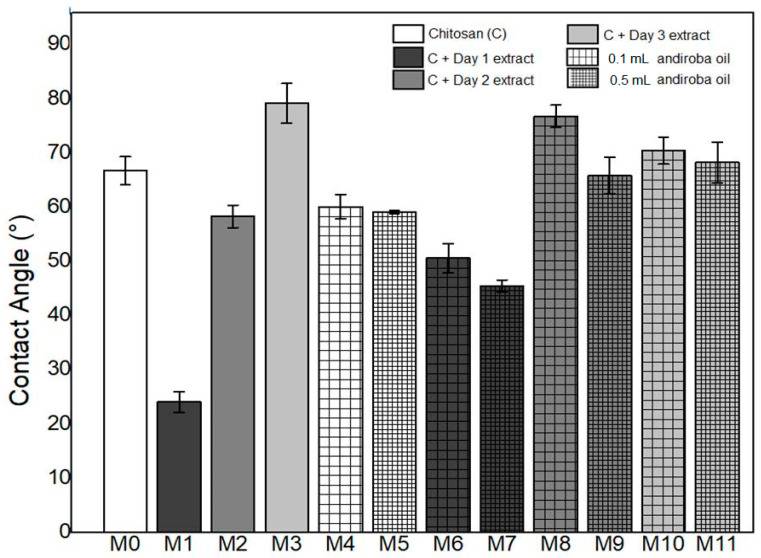
Comparison between the means of the values of the contact angles of the membranes.

The results of the measurements of the contact angles are presented in Figure 5.

From the value obtained for the contact angle, in Figure 5, it is possible to define the degree of wettability of a surface. At an angle less than 90°, a surface is defined as hydrophilic, and when it is more than 90°, a hydrophobic surface is obtained [21]. All synthesized membranes were defined as hydrophilic, but showed significant statistical differences among them (ANOVA, *p* < 0.005; F > F critic). The synthesis with an addition of green banana peel extract influenced the values of contact angles, being lower with extracts of the first and second decoction day and higher with the extract of the third decoction day, as compared to pure chitosan.

Franco et al. [8] found a similar result, indicating that the contact angle of the membranes with green banana peel extract from the first and third decoction days presented hydrophilic characteristics, but with a lower angle value in the sample prepared with the extract of the first day, suggesting that the presence of high concentration of extracted compounds modified the roughness of this membrane.

The concentration of andiroba oil prompted a decrease in the value of the contact angles, as shown in Figure 5, compared to the pure chitosan membrane—M0 (66.73 ± 2.56), those being M4 and M5 with values of 60.03 ± 2.25 and 59.06 ± 0.32, respectively. However, there was no statistically significant difference between the 0.1 mL and 0.5 mL andiroba oil concentrations.

Milk et al. [20] found that the addition of oil to chitosan membranes provided a decrease in the contact angle. However, a similar study by Silva et al. [22] found different results, demonstrating that the introduction of andiroba oil into the membrane composition confers greater hydrophobic power to the material.

From the results in Figure 5 it was estimated that the addition of the green banana peel extract had a greater influence on the contact angle value compared to the addition of andiroba oil alone. In spite of these differences, all synthesized membranes are candidates for application in epithelial lesions by their hydrophilic characteristics. Indeed, they presented the possibility of absorption of exudate from the wound and the maintenance of wound moisture, contributing to the process of cell regeneration in addition to minimizing pain due to the dryness of nerve endings.

### 3.4. SEM

**Figure 6:**
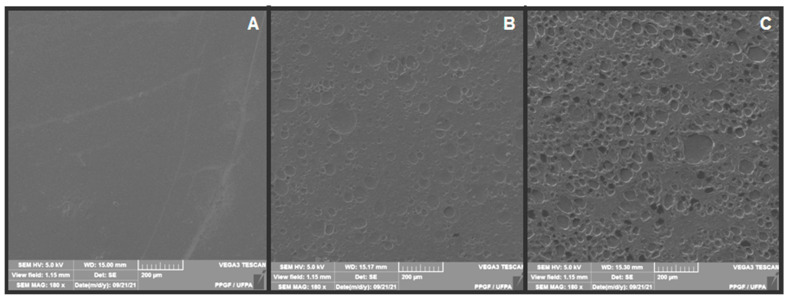
Micrographs obtained by SEM of membranes: (**A**) M0, (**B**) M4, (**C**) M5.

The micrographs obtained in the SEM analysis for the samples of plain chitosan (M0) and chitosan membranes with andiroba oil (M4 and M5) are observed in Figure 6. It should be noted that there was a difference between the samples, since M0, in Figure 6a, presented a smooth and homogeneous surface. On the other hand, the membranes with the addition of andiroba oil had an irregular surface compatible with the immiscibility between chitosan and oil, with elliptical structures distributed throughout the membrane. A quantitative increase of these rounded structures was observed in sample M5 (Figure 6c), and lower increase was found in sample M4 (Figure 6b), membranes with 0.5 mL and 0.1 mL of andiroba oil, respectively.

Similar results were found by Kimura et al. [19] with chitosan membranes containing andiroba oil, demonstrating ellipsoidal-shaped and discontinued structures associated with the formation of two phases in the matrix (lipid and polymer). Moreover, the number of oil drops increased as the andiroba oil concentration increased.

**Figure 7:**
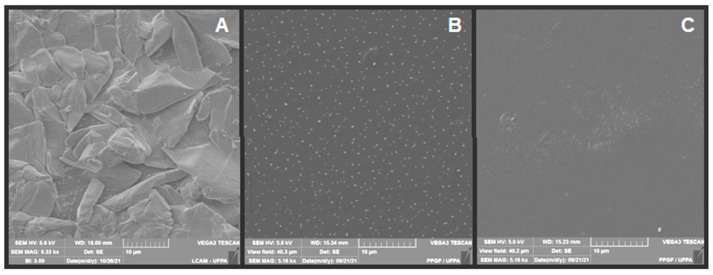
Micrographs obtained by SEM of membranes: (**A**) M1, (**B**) M2, (**C**) M3.

As for the membranes composed of chitosan and green banana peel extract without andiroba oil, it is observed in Figure 7 that, among the micrographs of samples synthesized with an extract from the first decoction day, Figure 7a demonstrates the presence of irregular aggregated and dispersed structures throughout the membrane, possibly due to the presence of phenolic compounds present in the extract. However, these structures are not visualized in the sample composed of the extract of the second decoction day, Figure 7b, revealing a surface with smaller homogeneous structures distributed throughout the membrane. The micrograph of the sample prepared with an extract from the third decoction day, Figure 7c, points to a more homogeneous surface than those in Figure 7a,b, but with the presence of some precipitates.

Franco et al. [8] found similar results, evidencing a plain chitosan membrane with dense, continuous, and homogeneous aspects, while chitosan with extracts of the first and third days of decoction had discontinuities and precipitates. These authors point out that the result of micrographs can be attributed to the presence of compounds from the green banana peel extract that structurally modified the chitosan matrix.

Similarly, a study by Kamel et al. [5], of chitosan membranes and green banana peel powder (0, 2, 5 and 10% by weight), obtained micrographs indicating uniform filling distributed within the chitosan matrix in the membranes with 2% of the powder. They affirmed that by increasing the powder concentration, there was irregular distribution with some aggregates.

Corroborating this study, Zhang et al. [18], studying chitosan films and banana peel extract, and exhibited micrographs with highly heterogeneous and porous structures, with a series of small white spots and a separation of phases, which were denser and more uniform with the decrease in the concentration of the extract.

**Figure 8:**
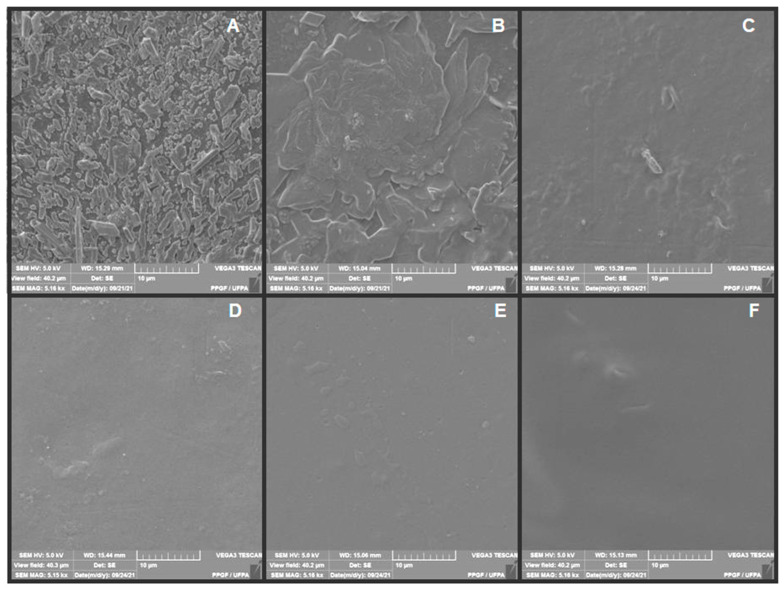
Micrographs obtained by SEM of membranes: (**A**) M6, (**B**) M7, (**C**) M8, (**D**) M9, (**E**) M10, (**F**) M11.

The small differences between the micrographs of the samples composed of different andiroba oil concentrations and green banana peel extract, demonstrated in Figure 8, indicate a greater influence of the extract on the micrography of the membrane surface.

The synthesized membranes with the green banana peel extract of the third decoction day, have smoother surfaces, with fewer aggregates, indicating a better dilution of the material. However, it is clear that this might not be suitable for the treatment of skin lesions due to the reduced number of tannins and other bioactive compounds. Therefore, there is a need to correlate this phenomenon with the results presented in the other tests performed, especially its association with andiroba oil.

### 3.5. XRD

**Figure 9:**
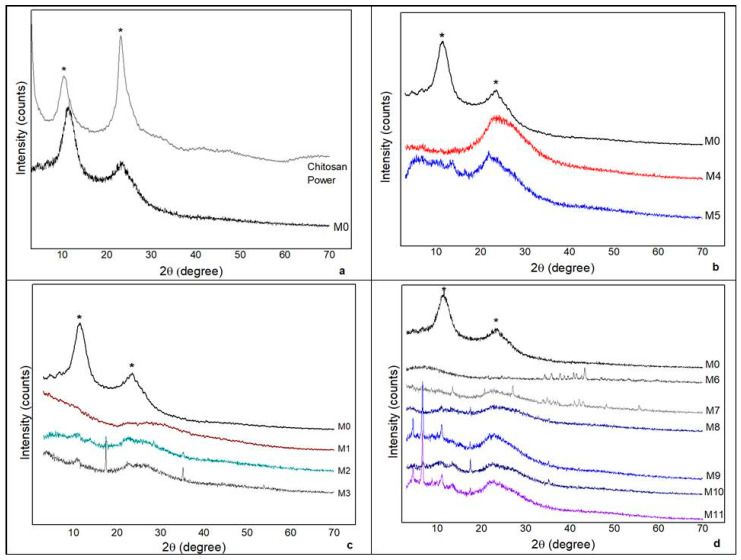
X-ray diffractogram. (**a**) Chitosan powder and pure chitosan membrane (M0). (**b**) M0 and chitosan membranes incorporated with andiroba oil (M4 and M5). (**c**) Chitosan membranes with green banana peel extract (M1, M2 and M3). (**d**) Chitosan membranes with green banana peel extract and/or andiroba oil. * Chitosan.

The diffractograms of chitosan powder and the membranes are presented in Figure 9.

Analyzing the diffraction chart presented in Figure 9a, two peaks with wide bands in 2θ are observed: 10.94° and 23.70° in M0 and 2θ: 10.55° and 23.37° in the powdered, as-received chitosan sample. When comparing the XRDs of chitosan powder with that of the synthesized chitosan membrane (M0), the figure demonstrates that the synthesis process did not eliminate the peaks. However, there was a change in their intensities. These peaks are described in the literature as characteristic of chitosan [23].

Chitosan has a semicrystalline profile caused by the formation of strong intramolecular and/or intermolecular hydrogen bonds between the amino and hydroxyl groups, which provide a certain organization to its structure [24].

The diffractogram presented in Figure 9b demonstrates that, by incorporating andiroba oil in the synthesis of the chitosan membrane, there is a greater amorphousness of M4 and M5 compared to M0. However, there is also a discrete peak (2θ: 23.54°) in M5.

The membranes synthesized with extract from the first day of decoction (M1) showed no peaks. However, two discrete peaks were visualized in M2 (2θ: 17.48° and 35.28°). Their intensification in M3 (2θ: 17.45° and 35.23°) is shown in Figure 9c.

These results coincide with those of another study [18], in which the incorporation of banana peel powder into the chitosan membrane showed gradual disappearance of the peak, suggesting that the incorporation of the powder led to a decline in membrane crystallinity.

According to Kadam and Lele [25], the interaction between the chitosan molecule and banana peel powder alters the original arrangement of the polymer, resulting in the formation of an amorphous complex.

The diffractograms of chitosan membranes with green banana peel extract and andiroba oil presented in Figure 9d indicate amorphous structures, with little difference between those that do not contain green banana peel extract. Therefore, the absence of crystallinity might be related to the presence of the constituents of the plant components present in both the extracts and the oils.

As reported by Barrioni et al. [26], predominantly amorphous materials are preferred for biomedical use because they present greater bioactivity when compared to crystalline materials. Therefore, based on the results disclosed in this study, the membranes synthesized with green banana peel extract from the third decoction day do not have beneficial features for use as biomaterial, since the lower the concentration of the extract, the greater the crystallinity of the material.

### 3.6. DSC

**Figure 10:**
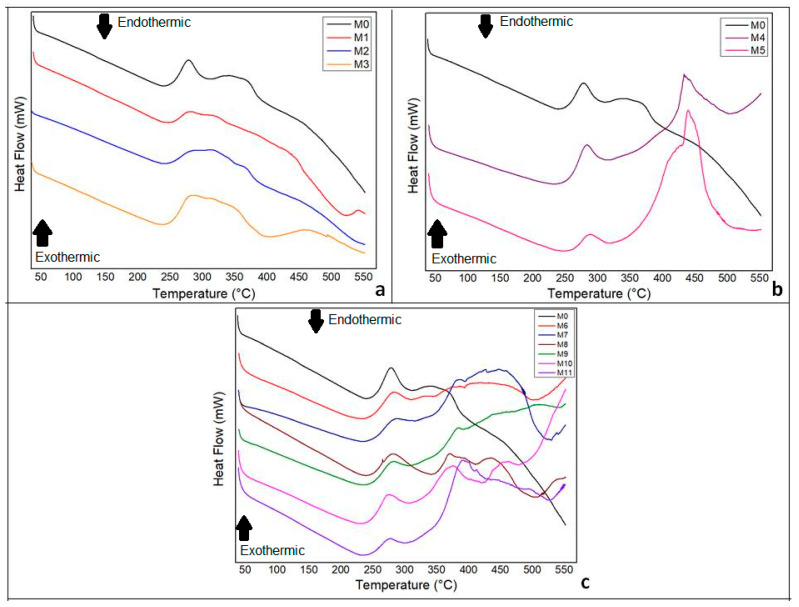
DSC analysis (second heat) of chitosan biomembranes. (**a**) Chitosan pure membrane (M0) and membranes with green banana peel extract (M1, M2 and M3) (M0). (**b**) M0 and chitosan membranes incorporated with andiroba oil (M4 and M5). (**c**) Chitosan membranes with green banana peel extract and/or andiroba oil.

The DSC thermograms of chitosan membranes synthesized with and without green banana peel extract and andiroba oil are presented in Figure 10.

In Figure 10a, the exothermic peak of plain chitosan membrane (M0) is observed at 279 °C, with a decrease in the intensity of these peaks in chitosan membranes with green banana peel extract. It was found that higher concentrations of tannic acids in the extract increased its degradation temperature.

The second thermal event, around 270–290 °C was attributed to structural decomposition and was commonly related to the degradation of glucosamine units in the chitosan structure. The deviation of this event to lower temperatures confirms the reduction of its structural stability [26], which we did not observe in this study.

By incorporating andiroba oil in the synthesis of membranes, the thermogram, Figure 10b, reveals the appearance of a new exothermic peak between 433–439 °C, previously absent in the pure chitosan membrane (M0). The greater the amount of oil added, the higher the intensity of the peak. This fact might be explained by the hydrophobic character of the andiroba oil and also by the reduction of enthalpy related to the evaporation of the volatile compounds.

The addition of both plant constituents, green banana peel extract and andiroba oil, influenced the emergence of peaks in different degrees from 370 °C when compared with plain chitosan membrane, as shown in Figure 10c.

## 4. Summary and Conclusions

In this study, membranes were prepared with chitosan, with and without the addition of green banana peel extract and incorporation with andiroba oil. These membranes were evaluated by macroscopic and morphological analyses. The surfaces of the membranes were characterized by swelling and moisture tests, contact angles, XRD and DSC.

- There was superiority in the percentage of moisture in the membranes composed of green banana peel extract and a higher percentage of swelling in the chitosan membranes with andiroba oil, demonstrating inverse proportionality between the swelling and the moisture of the synthesized membranes.
- The synthesized membranes were hydrophilic. However, the values of the contact angles were lower with green banana peel extracts and higher concentrations of andiroba oil.
- The XRD indicated amorphous material with discrete peaks in some samples and may be related to the presence of the constituents of the plant components present in both the extracts and the oils.
- The DSC demonstrated that the addition of the plant constituents, green banana peel extract and andiroba oil, influenced by the crystallinity of the material.
- Membranes composed of chitosan, green banana peel extract, and andiroba oil have characteristics to make up a biomaterial to treat epithelial lesions, gathering essential properties such as absorption capacity and fluid retention, cellular adhering facilitated by decreased crystallinity and thermal degradation.
- Therefore, the concentrations of the constituents should be adequately studied. In addition, it is necessary to carry out biological tests in the laboratory to evaluate their effectiveness in tissue regeneration.

## Figures and Tables

**Table 1:** Description of the materials used in the synthesis of membranes.

Material	Description	Brand/Supplier
Commercial chitosan	CAS: 9012–76–4) (medium molecular weight-190,000–310,000 Da and deacetylation degree in the range 75–85%)	Sigma Aldrich Laboratory (Sao Paulo, Brazil)
Glacial acetic acid	Pure, molecular weight 60.05 g/mol	Scientific Exodus (Sao Paulo, Brazil)
Sodium hydroxide solution 5%	Molecular weight 40 g/mol	Scientific Exodus (Sao Paulo, Brazil)
Andiroba oil Species: *Carapa guianensis*	Acidity level < 15.0% weight, density 0.9261 g/mL to 25 °C, iod index 55–80 gI_2_/100 g, melting point 22 °C, peroxide content < 10.0 (10 meq O_2_/kg), saponification index 190–210 mg KOH/g, insaponifiable matter (bioactive) 3–5%	Amazon Oil (Belém, Pará, Brazil)
Green banana peel	Species *Musa* spp., category 1 maturation Von Loeseck [15].	Cultivated in the municipality of Santo Antônio do Tauá, Pará, Brazil.

**Table 2:** Description of the acronyms and composition of the synthesized membranes.

Acronym	Composition
M0	Chitosan + distilled water
M1	Chitosan + green banana peel extract from the 1st day
M2	Chitosan + green banana peel extract from the 2nd day
M3	Chitosan + green banana peel extract from the 3rd day
M4	Chitosana + distilled water + 0.1 mL andiroba oil
M5	Chitosana + distilled water + 0.5 mL andiroba oil
M6	Chitosan + green banana peel extract from the 1st day + 0.1 mL andiroba oil
M7	Chitosan + green banana peel extract from the 1st day + 0.5 mL andiroba oil
M8	Chitosan + green banana peel extract from the 2nd day + 0.1 mL andiroba oil
M9	Chitosan + green banana peel extract from the 2nd day + 0.5 mL andiroba oil
M10	Chitosan + green banana peel extract from the 3rd day + 0.1 mL andiroba oil
M11	Chitosan + green banana peel extract from the 3rd day + 0.5 mL andiroba oil

**Table 3:** Comparison of the visual macroscopic aspect of chitosan membranes and green banana peel extract obtained in the study.

Visual Appearance	M0	M1	M2	M3	M4	M5	M6	M7	M8	M9	M10	M11
Colour	0	3	2	1	0	0	2	2	1	1	1	1
Uniformity	3	2	2	2	1	1	2	2	3	3	3	3
Transparency	3	0	2	2	2	2	1	1	1	1	2	2
Bubbles/Cracks	1	1	1	1	1	1	1	2	1	1	1	2
Brittle aspect	1	0	0	0	0	0	0	0	0	0	0	0
Flexibility	2	3	3	3	3	3	3	3	3	3	3	3
Detachment of the support	2	2	2	2	3	3	3	3	3	3	3	3

0-absence; 1-little; 2-medium; 3-intense.

**Table 4:** Percentage of swelling in water and PBS solution of pH 7.2 after the period of 24 h and percentage of moisture of the synthesized membranes.

Sample		M0	M1	M2	M3	M4	M5	M6	M7	M8	M9	M10	M11
Swelling (%)	Water	72.4 ± 15.8	11.1 ± 9.9	49.3 ± 5.0	81.3 ± 16.7	133.3 ± 68.3	135.0 ± 18.5	32.3 ± 2.9	34.6 ± 19.8	77.8 ± 6.4	72.9 ± 39.9	98.7 ± 49.7	108.3 ± 19.6
	PBS	66.6 ± 16.7	22.9 ± 14.8	53.6 ± 23.9	84.6 ± 44.4	82.7 ± 26.1	71.1 ± 15.4	32.3 ± 9.2	43.4 ± 30.2	51.8 ± 13.4	47.8 ± 19.2	68.8 ± 28.9	69.0 ± 32.2
Moisture (%)		21.2 ± 1.2	23.1 ± 1.1	21.3 ± 0.3	22.2 ± 2.1	14.7 ± 1.9	14.9 ± 0.9	17.6 ± 7.0	24.9 ± 3.8	16.9 ± 3.0	14.9 ± 3.8	18.0 ± 2.4	23.8 ± 3.7

## Data Availability

Not applicable
